# Strong reproductive barriers in a narrow hybrid zone of West-Mediterranean green toads (*Bufo viridis *subgroup) with Plio-Pleistocene divergence

**DOI:** 10.1186/1471-2148-10-232

**Published:** 2010-07-29

**Authors:** Caroline Colliard, Alessandra Sicilia, Giuseppe Fabrizio Turrisi, Marco Arculeo, Nicolas Perrin, Matthias Stöck

**Affiliations:** 1 Department of Ecology and Evolution, Biophore, University of Lausanne, CH-1015 Lausanne, Switzerland; 2 Dipartimento di Biologia Animale, University of Palermo, Via Archirafi, 18, 90123 Palermo, Italy; 3 University of Catania, CUTGANA, Section of Nature Reserve Management, via Terzora 8, 95027 San Gregorio di Catania, Catania, Italy

## Abstract

**Background:**

One key question in evolutionary biology deals with the mode and rate at which reproductive isolation accumulates during allopatric speciation. Little is known about secondary contacts of recently diverged anuran species. Here we conduct a multi-locus field study to investigate a contact zone between two lineages of green toads with an estimated divergence time of 2.7 My, and report results from preliminary experimental crosses.

**Results:**

The Sicilian endemic *Bufo siculus *and the Italian mainland-origin *B. balearicus *form a narrow hybrid zone east of Mt. Etna. Despite bidirectional mtDNA introgression over a ca. 40 km North-South cline, no F_1 _hybrids could be found, and nuclear genomes display almost no admixture. Populations from each side of the contact zone showed depressed genetic diversity and very strong differentiation (F_ST _= 0.52). Preliminary experimental crosses point to a slightly reduced fitness in F_1 _hybrids, a strong hybrid breakdown in backcrossed offspring (F_1 _x parental, with very few reaching metamorphosis) and a complete and early mortality in F_2 _(F_1 _x F_1_).

**Conclusion:**

Genetic patterns at the contact zone are molded by drift and selection. Local effective sizes are reduced by the geography and history of the contact zone, *B. balearicus *populations being at the front wave of a recent expansion (late Pleistocene). Selection against hybrids likely results from intrinsic genomic causes (disruption of coadapted sets of genes in backcrosses and F_2_-hybrids), possibly reinforced by local adaptation (the ranges of the two taxa roughly coincide with the borders of semiarid and arid climates). The absence of F_1 _in the field might be due to premating isolation mechanisms. Our results, show that these lineages have evolved almost complete reproductive isolation after some 2.7 My of divergence, contrasting sharply with evidence from laboratory experiments that some anuran species may still produce viable F_1 _offspring after > 20 My of divergence.

## Background

One key question in evolutionary biology deals with the mode and rate at which reproductive isolation accumulates during allopatric speciation [for overview: [1]]. Johns and Avise [2] estimated the average mitochondrial DNA (mtDNA)-based genetic distance between congeneric species in amphibians to be > 7.0 My, suggesting absence of natural hybridization in taxa of that age. A few major results on intrinsic reproductive isolation in anurans come from artificial hybridization experiments. Sasa *et al. *[3] reported hybrid sterility or inviability in 46 frog species to be positively correlated with Nei's genetic distance (allozymes). Measuring albumin distances among 50 species pairs, Wilson *et al. *[4] showed that frogs could still produce viable hybrids with an average immunological distance of 7.4% (= ca. 21 My). Using Blair's [5] crossing experiments in *Bufo*, Malone & Fontenot [6] showed the hatching success, the number of larvae produced, and the percentage of tadpoles reaching metamorphosis to be inversely related with genetic divergence, some metamorphosing offspring being still produced with a distance of 8% (mtDNA). All of these laboratory data suggest that reproductive isolation increases gradually with phylogenetic distance, presumably driven by complex genomic processes rather than by a few speciation genes, and that very large time scales (in the order of tens of millions of years) are required to achieve hybrid infertility or inviability.

Under natural conditions, however, reproductive isolation could arise much earlier than detected in the laboratory. In frogs, as in many other taxa, "surveys of natural hybrid zones (...) in the field are needed to complement laboratory-based studies to establish the significance and strength of specific barriers in nature" [7]. Little is known about secondary contact in allopatrically diverged lineages of anurans, where reproductive isolation may quickly arise as a result of reinforcement [8], in addition to genetic drift and local adaptation. Extant studies of contact zones in anurans have mostly focused on hybrid fitness [9,10] or on mechanisms of pre- or post-mating isolation [9-15]. Such studies classically relied on allozymes [e.g. [9,11-13]] or (more recently) nuclear and mitochondrial DNA markers [e.g. [10,14,15]], but often lack any molecular-based estimates of divergence times, which are at best inferred from geological information. Some phylogeographic studies include molecular-based estimates of divergence time [e.g. [16-20]], but very few have combined such estimates with multi-locus transect approaches to infer the time required to reach reproductive isolation in natural contexts [e.g. [8,21,22]].

**Figure 1:**
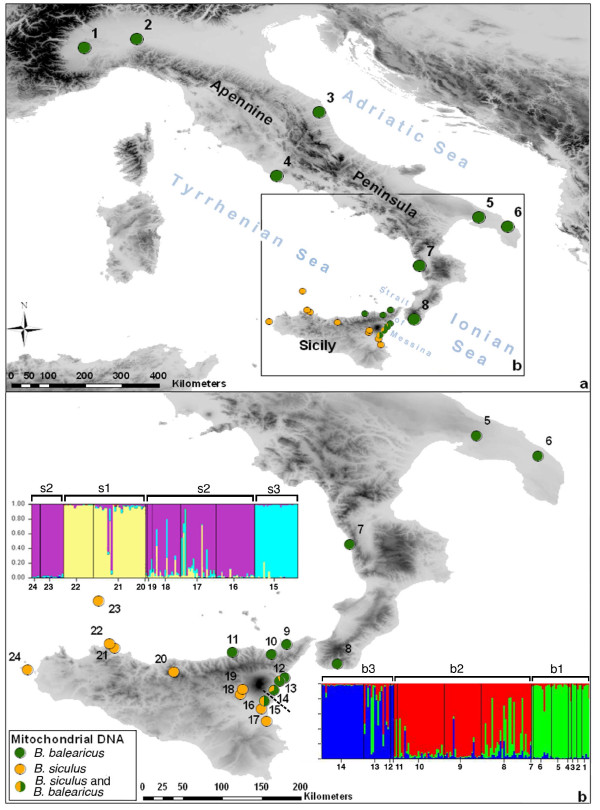
**Geographical overview of the study region (with population genetic clustering)**. a. Sampling sites across the entire study area with major mtDNA haplotype groups. Orange symbols: *B. siculus*; green symbols: *B. balearicus*. Haplotypes from both lineages were detected in three localities (pop. 13 to 15) east of Mt Etna with ratios shown as pie charts. b. Sampling sites of southern Apennine Peninsula, Sicily and two off-coast islands with mtDNA haplotype groups. Also plotted are assignment probabilities based on STRUCTURE analyses for all *B. siculus *individuals (K = 3, left) and all *B. balearicus *individuals (K = 3, right). For clusters (*balearicus*: b1 to b3, *siculus*: s1 to s3) see text; the dashed line (in b) between localities 14 and 15 refers to the region where the abrupt change for the nuclear markers is observed.

The current study focuses on Palearctic green toads [*Bufo viridis *subgroup, [18]]. After range-wide phylogeographic analyses, secondary contact zones of clades were predicted [18,23], in which possible hybridization can be examined using fast evolving molecular markers. To do this, we recently developed microsatellites for two West-Mediterranean species [24]: *B. balearicus *(Boettger 1880; Peninsular Italy, north-eastern Sicily, Corsica, Sardinia, Balearic Islands), and *B. siculus *[[23]; endemic to Sicily, Figure 1]. Using a Bayesian-coalescence approach (mtDNA control region and 16 S rRNA), divergence time for the two species was estimated to late Pliocene (2.7 My), with a range from the early Pliocene (4.9 My) to Pleistocene (1.1 My) [23]. A single record of Italian mainland-origin *B. balearicus *in north-eastern Sicily [18] suggests their recent (late Pleistocene) invasion into Sicily, where they may secondarily meet the endemic *B. siculus*.

In this work, we combined mitochondrial and nuclear intronic sequences with multilocus microsatellite markers to examine (i) whether *B. siculus *and *B. balearicus *meet each other in north-eastern Sicily, (ii) if so, whether these two closely related species hybridize, and (iii) in such a case, what are the patterns of hybridization. In parallel, we conducted limited and preliminary experimental crosses to help interpreting field data.

## Results

### Nuclear and mitochondrial DNA sequences and mitotyping

**Figure 2:**
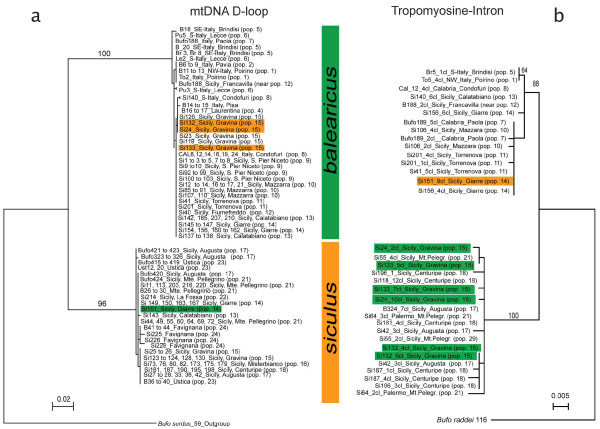
**Phylogenetic trees of mitochondrial and nuclear markers**. Maximum likelihood trees based on 577 bp of the mitochondrial d-loop (left, a), and of several clones (cl.) obtained from 580 bp of an intron of tropomyosine, situated between exons 5 and 6 (right, b). Specimen number (sometimes several with same haplotype and locality) is followed by locality information and population number (as in Figure 1 and Table 1). Individuals highlighted in colour possess a d-loop haplotype group of one species but tropomyosine alleles from the opposite species.

Both of the phylogenetic trees built from mitochondrial (D-loop) and nuclear (Tropomyosine intron) DNA sequences show two highly homogeneous and strongly distinct clades (Figure 2), corresponding to *B. balearicus *and *B. siculus*. The tropomyosine tree displays a clear geographic pattern: the *balearicus *clade includes all individuals from mainland Italy (populations 1 to 8, Figure 1, Table 1) and north-eastern Sicily, southwards to population 14 (east coast), while the *siculus *clade includes individuals from western and southern Sicilian populations, from population 15 (East coast) south-westwards. This pattern points to a very narrow contact zone separating populations 14 and 15, between the Mount Etna and the Ionian coast (Figure 1b).

**Table 1:** Localities, major regions of origin, geographic coordinates (degrees) and number of green toad samples from larvae, subadults and adults

Localities	Region	Longitude	Latitude	Individuals	Tadpoles	Subadults	Adults	Males	Females
**1 **(Poirino)	IP	7.846	44.920	5	0	0	5	-	-
**2 **(Pavia)	IP	9.142	45.155	3	0	0	3	-	-
**3 **(Morrovalle)	IP	13.586	43.280	4	0	0	4	-	-
**4 **(Laurentina)	IP	12.548	41.645	2	0	0	2	-	-
**5 **(Brindisi)	IP	17.475	40.586	10	9	0	0	-	-
**6 **(Lecce)	IP	18.174	40.353	11	11	0	0	-	-
**7 **(Paola)	IP	16.033	39.35	1	0	0	1	0	1
**8 **(Condofuri)	IP	15.894	37.985	30	19	1	10	9	-
**9 **(San Pier Niceto)	Sicily	15.318	38.211	22	12	0	10	10	-
**10 **(Mazzarrà)	Sicily	15.138	38.096	28	7	0	21	20	-
**11 **(Torrenova)	Sicily	14.699	38.118	2	0	0	2	-	2
**12 **(Fiumefreddo)	Sicily	15.23	37.789	2	0	0	2	1	1
**13 **(Calatabiano)	Sicily	15.243	37.796	17	0	1	16	14	2
**14 **(Giarre)	Sicily	15.174	37.691	25	0	0	25	21	4
**15 **(Gravina)	Sicily	15.063	37.561	26	0	4	22	21	1
**16 **(Misterbianco)	Sicily	15.022	37.476	24	24	0	0	-	-
**17 **(Augusta)	Sicily	15.08	37.334	21	0	8	13	9	3
**18 **(Centuripe)	Sicily	14.788	37.643	17	0	0	17	13	4
**19 **(Bronte)	Sicily	14.813	37.698	3	0	0	3	1	1
**20 **(Monte Carbonara)	Sicily	14.025	37.894	1	0	0	1	1	-
**21 **(Monte Pellegrino)	Sicily	13.352	38.17	31	0	1	30	-	-
**22 **(La Fossa)	Sicily	13.292	38.213	18	0	0	18	17	1
**23 **(Ustica)	Island	13.172	38.701	15	5	0	10	-	-
**24 **(Favignana)	Island	12.36	37.921	5	4	0	1	0	-

**Total**				**323**	**91**	**15**	**216**	**137**	**20**

Sexes were only determined in a subset of adults (IP: Italian Peninsula).

The mtDNA clades also show a clear geographic signal, with however, some overlap. Populations from mainland Italy (pop. 1 to 8) and north-eastern Sicily (pop. 9 to 12) present only *balearicus *haplotypes, and populations from western and southern Sicily (pop. 16 to 24) only *siculus*, but haplotypes from both clades are found in populations 13 to 15, around the contact zone identified with tropomyosine.

These phylogenetic trees also provide evidence for past hybridization, as revealed by cytonuclear disequilibria (see highlighted individuals in Figure 2): one individual from pop. 14 possesses *balearicus *tropomyosine alleles but a *siculus *mtDNA-haplotype, while three individuals from pop. 15 present *siculus *tropomyosine alleles but *balearicus *mtDNA haplotypes.

These patterns of mitochondrial distribution were widely confirmed by larger-scale mitotyping (Figure 1). All populations on the Apennine Peninsula (pop. 1 to 8) and four populations (pop. 9 to 12) from the North-East of Sicily presented only *B. balearicus *haplotypes. All populations from western and southern Sicily (pop. 16 to 22) and the two islands off the coast of western Sicily (pop. 23, 24) presented only *B. siculus *haplotypes. In the three populations east of Mount Etna (pop. 13 to 15), both *B. balearicus *and *B. siculus *haplotypes were present, with a marked north-south cline (Table 2): The frequency of *balearicus *haplotypes declined from 93.75% in Calatabiano (pop. 13) to 68% in Giarre (pop. 14) and 50% in Gravina (pop. 15), down to 0% in Misterbianco (pop. 16).

**Table 2:** Percentage of potential hybrids detected in populations where hybrids are expected or likely to occur

		MtDNA		Nuclear DNA		Hybrids, based on		
**Population**	**N**	***B. balearicus***	***B. siculus***	**pure *B. balearicus***	**pure *B. siculus***	**NewHybrids**	**Diagnostic alleles**	**Cytonuclear disequilibrium**

**13 **(Calatabiano)	16	15	1	14	0	2	1	1
	100%	93.75%	6.25%	87.50%	0%	12.50%	6.25%	6.25%

**14 **(Giarre)	25	17	8	25	0	0	2	8
	100%	68%	32%	100%	0%	0%	8%	32%

**15 **(Gravina)	26	13	13	0	26	0	4	13
	100%	50%	50%	0%	100%	0%	15.38%	50%

**16 **(Misterbianco)	24	0	24	0	24	0	0	0
	100%	0%	100%	0%	100%	0%	0%	0%

**18 **(Centuripe)	17	0	17	0	15	2	2	0
	100%	0%	100%	0%	88.24%	11.76%	11.76%	0%

MtDNA: Numbers and percentages of individuals containing either *B. balearicus *or *B. siculus *mtDNA. Nuclear DNA: Numbers and percentages of individuals assigned by STRUCTURE to either *B. balearicus *or to *B. siculus*. Hybrids based on: potential hybrids detected using a) NEWHYBRIDS software, b) diagnostic microsatellite alleles or c) cyto-nuclear disequilibrium.

### Autosomal microsatellites and population-genetics analyses

There was no evidence for allelic dropout from any locus in any population. Null alleles at low frequencies were detected (and corrections performed) in one population each for loci C203 (pop. 14) and D105 (pop. 13), and in two populations each for loci C218 (pop. 6, 22), C223 (pop. 18, 21) and D5 (pop. 17, 18). Tests for linkage disequilibrium between loci (after sequential Bonferroni corrections) revealed four significant combinations (Bcal μ10 × C203 for pop. 9, D5 × Bcal μ10 for pop. 15, C223 × Bcal μ10 for pop. 18, D105 × C205 for pop. 23). A few *B. siculus *populations showed some heterozygote deficit (Additional file 1), presumably due to sampling design (substructures may arise when pooling tadpoles or adults from several nearby ponds).

**Figure 3:**
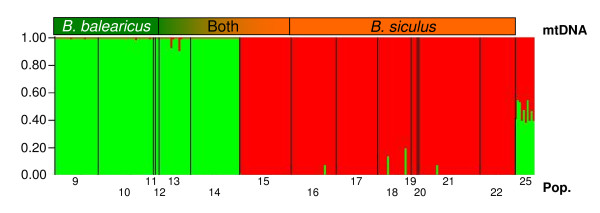
**Genotype-based assignment of Sicilian green toads based on Bayesian cluster analyses**. Bar plots from the program Structure based on seven microsatellite markers for green toads coming from Sicily for K = 2. Population 25 represents F_1_-hybrids coming from a laboratory cross between one female *B. balearicus *(pop. 11) with a male *B. siculus *(pop. 22).

Bayesian clustering assignment using STRUCTURE [25] largely confirmed the nuclear information from Tropomyosine. All populations from Sicily were clearly grouped into two clusters [K = 2; [26]] corresponding to *B. balearicus *and *B. siculus *gene pools respectively (Figure 3). All individuals from populations 9 to 14 were assigned to *B. balearicus*, while all individuals from populations 15 to 24 were assigned to *B. siculus*. Ten F_1_-hybrids from an experimental cross between a female *balearicus *(pop. 11) and a male *siculus *(pop. 22) were correctly assigned a 50% probability of belonging to either *balearicus *or *siculus *(pop. 25). Surprisingly, the two populations north and south of the contact zone (pop. 14 and 15) did not show any sign of hybridization or gene flow, despite harboring mtDNA from both clades. All individuals from population 14 were assigned with a 100% probability to *balearicus*, and all individuals from the population 15 with 100% probability to *siculus*. As a matter of fact, potential hybrids appear very few (altogether four individuals with assignment probabilities lower than 90% to either parental species), and largely backcrossed (assignment probabilities to the alternative parental species lower than 25%).

This pattern was confirmed by NEWHYBRIDS [27], which, when including all Sicilian populations, correctly assigned all experimental crosses as F_1_-hybrids, and identified four wild-caught individuals as possible F_2_-hybrids (two each from pop. 13 and 18, details in Additional file 2). When focusing on populations where hybrids occurred or were likely to do so (pop. 12 to 16 and 18), while pre-assigning pop. 9 to 11 and 17 as pure *B. balearicus *and *B. siculus*, respectively, no nuclear hybrids were detected. Finally, diagnostic alleles also suggested faint signs of past hybridizations (Additional file 3). We found *B. siculus *alleles in three individuals assigned as *B. balearicus *using STRUCTURE (one from pop. 13 and two from pop. 14), and *B. balearicus *alleles in six individuals assigned as *B. siculus *(four in pop. 15 and two in pop. 18). All of these analyses concur to suggest limited events of nuclear introgression.

In order to fine-tune our analysis of potential gene flow, we performed separate STRUCTURE analyses for *B. balearicus *and *B. siculus *populations. Substructure within *B. balearicus *was best explained with K = 3 (Figure 1b). Cluster b1 contained individuals from mainland Italy, cluster b2 individuals from north-eastern Sicilian populations, and cluster b3 individuals from populations close to the contact zone. Interestingly population 8 (tip of Calabria) showed admixture of mainland Italy and Sicilian genotypes. Substructure within *B. siculus *was similarly best explained with K = 3 (Figure 1b). Cluster s2 contained individuals from the vast majority of populations (including off coast islands, pop. 23 and 24), except for populations 21 and 22 (north-western coast of Sicily, cluster s1) and population 15 at the contact zone (cluster s3).

**Figure 4:**
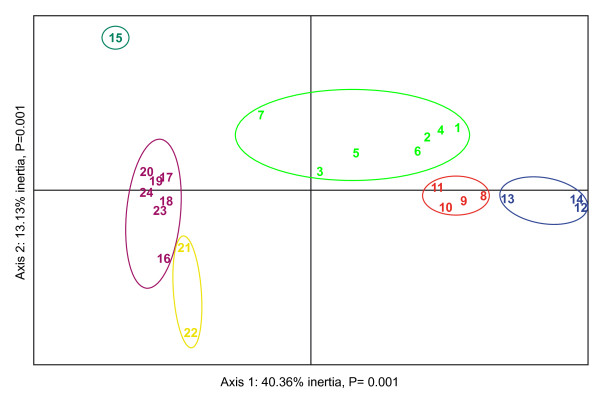
**Principal component analysis based on pairwise F_ST _over all populations**. Both axes are significant (P < 0.001). Samples are encoded as in Table 1. Colored ellipsoids correspond to clusters shown in Figure 1b and were only drawn for better visualization.

Hence, in both species, the populations close to the hybrid zone (showing coexistence of mtDNA haplotypes) form a cluster of their own. However, this pattern is clearly not generated by nuclear gene flow between the two species. Indeed, from pair-wise F_ST _values, the strongest differentiation (F_ST _= 0.52, Additional file 4) actually occurs between the two populations (pop. 14 and 15) from each side of the contact zone, as compared to an average value of 0.32 between allospecific populations (and 0.18 between conspecific populations). This unexpected result was confirmed by a principal component analysis [PCAGEN; [28]] aimed at extracting factors maximizing genetic differentiation among populations (Figure 4). Two factors turn out to be significant, explaining respectively 40.4 and 13.1% of the total differentiation (F_ST_). The first one accounts for the contrast between *B. siculus *(left) and *B. balearicus *(right). The three *B. balearicus *clusters identified with STRUCTURE differentiate along this axis, with lowest values for b1 (mainland Italy, pop. 1 to 7), and highest values for b3 (pop. 12 to 14, close to the contact zone). The three *B. siculus *clusters differentiate mostly on the second factor, with lowest values for s1 (north-western coast, pop. 21 and 22) and highest values for s3 (pop. 15, close to the contact zone).

The spread of clusters correlates with geography, which translates into some isolation by distance. The relationship between genetic differentiation and geographic distance is strong and significant in both species when dropping the three populations (pop. 13 to 15) at the contact zone (r = 0.81, R^2 ^= 66%, p = 0.0026 for *B. balearicus*, and r = 0.41, R^2 ^= 17%, p = 0.035 for *B. siculus*), but drastically reduced when including these three populations, due to strong differentiation over short geographic distances (r = 0.23, R^2 ^= 8.72%, p = 0.25 for *B. balearicus*, and r = 0.21, R^2 ^= 4.65%, p = 0.47 for *B. siculus*).

This enhanced differentiation between populations close to the contact zone correlates with increased genetic drift and loss of diversity. Genetic diversity in *B. balearicus *populations decreases from Hs = 0.74 in mainland Italy (pop. 5 and 6) to 0.54 in Calabria and Northern Sicily, down to 0.38 at the contact zone (pop. 14). Similarly (though to a lesser extent), genetic diversity in *B. siculus *populations decreases from Hs = 0.75 South and West of the Mount Etna (pop. 17 and 18) to 0.62 in populations closer to the contact zone (pop. 15 and 16; Additional file 1, see also [24] for representative populations).

### Crossing experiments

**Figure 5:**
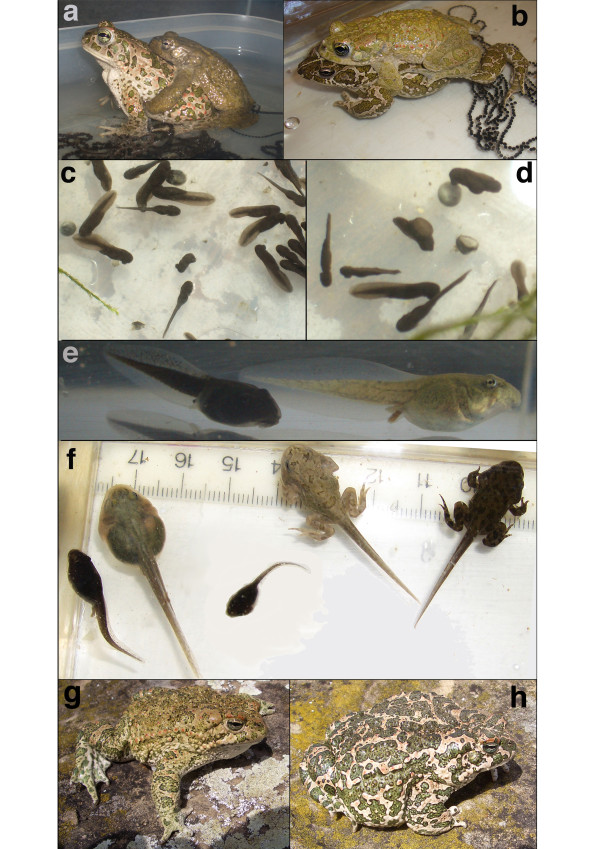
**Crosses of green toads from Sicily**. **a**: Cross *B. balearicus *female × *B. siculus *male; **b**: reciprocal cross *B. siculus *× *B. balearicus*; **c-h **F_1_-offspring from cross shown in **a**; **c**-**d**: offspring in the age of seven days, showing dead and malformed embryos and tadpoles in comparison with apparently normally developing ones; **e**: about one-months old normal tadpole (left) in comparison with leucistic "large" tadpole (right); **f**: in the age of two months (from left to right): retarded tadpole, "giant" leucistic tadpole with developmental arrest, malformed dwarfed tadpole, leucistic tadpole that turned later out to be incapable of metamorphosis, apparently normally metamorphosing tadpole; **g**: adult, two-year-old F_1_-male; **h**: adult, two-year-old F_1_-female. Photographs: M. Stöck.

From an F_1_-cross *B. balearicus *x *B. siculus *obtained in spring 2007, about 80% offspring were viable and developed normally (Table 3). The remaining 20% did not hatch or produced malformed, dwarfed and/or leucistic larvae (Figure 5c-f). Most of these died at early stages or during metamorphosis (four-legged stage), and a few ones survived as never-metamorphosing "giant" tadpoles (Figure 5f). The reciprocal cross (*B. siculus *x *B. balearicus*) showed much lower survival after metamorphosis (Table 3).

**Table 3:** Crossing experiments

Cross	Cross type	Female	Species/hybrid	Male	Species/hybrid	N of available tadpoles (at day 7 after spawning)	Ca.% Estimated hatching success	N of tadpoles (2 months after spawning)	% Survival (2 months after spawning)	Remarks	Survival at day 40 after metamorphosis
**1**	F_1_	Si41	*balearicus*	Si11	*siculus*	200	> 80	158	79	malformations, some dwarfed or leucistic larvae	150

**2**	F_1_	Si337	*balearicus*	Si334	*siculus*	100	> 80	98	98	died through technical accident	N.A.

**3**	F_1_	Si335	*siculus*	Si336	*balearicus*	100	> 80	93	93	-	4

**4**	Backcross (F_1 _× parental species)	Si337	*balearicus*	F_1 _Cross 13	F_1 _(bal. × sic.)	100	> 80	84	84	67 (big), 17 (small)	2

**5**	Backcross (F_1 _× parental species)	Si335	*siculus*	F_1 _Cross 13	F_1 _(bal. × sic.)	100	> 80	91	91	-	0

**6**	Backcross (F_1 _× parental species)	F_1 _Cross 11	F_1 _(bal. × sic.)	Si334	*siculus*	100	ca. 50	1	1	-	0

**7**	Backcross (F_1 _× parental species)	F_1 _Cross 11	F_1 _(bal. × sic.)	Si336	*balearicus*	28	ca. 30	24	85.7	1 color mutant, all dead (at day 116 after spawning)	0

**8**	F_2 _(F_1 _× F_1_)	F_1 _Cross 11	F_1 _(bal. × sic.)	F_1 _Cross 13	F_1 _(bal. × sic.)	100	ca. 20	0	0	all dead (at day 17 after spawning)	0

**Control1**	Intra-specific mating	Ky109	*turanensis*	Ky103	*turanensis*	50*	> 80	48	94	-	47

**Control2**	Intra-specific mating	Ky100	*pewzowi*	Ky87	*pewzowi*	50*	> 80	47	90	-	45

Cross number, cross type, sexes, species, estimated hatching success, numbers of tadpoles at 7 day and two months after spawning, percentage of survival two months after spawning, remarks and survival at day 40 after metamorphosis for each cross. *Note, in controls from two related green toad species, only 50 tadpoles were further raised.

**Figure 6:**
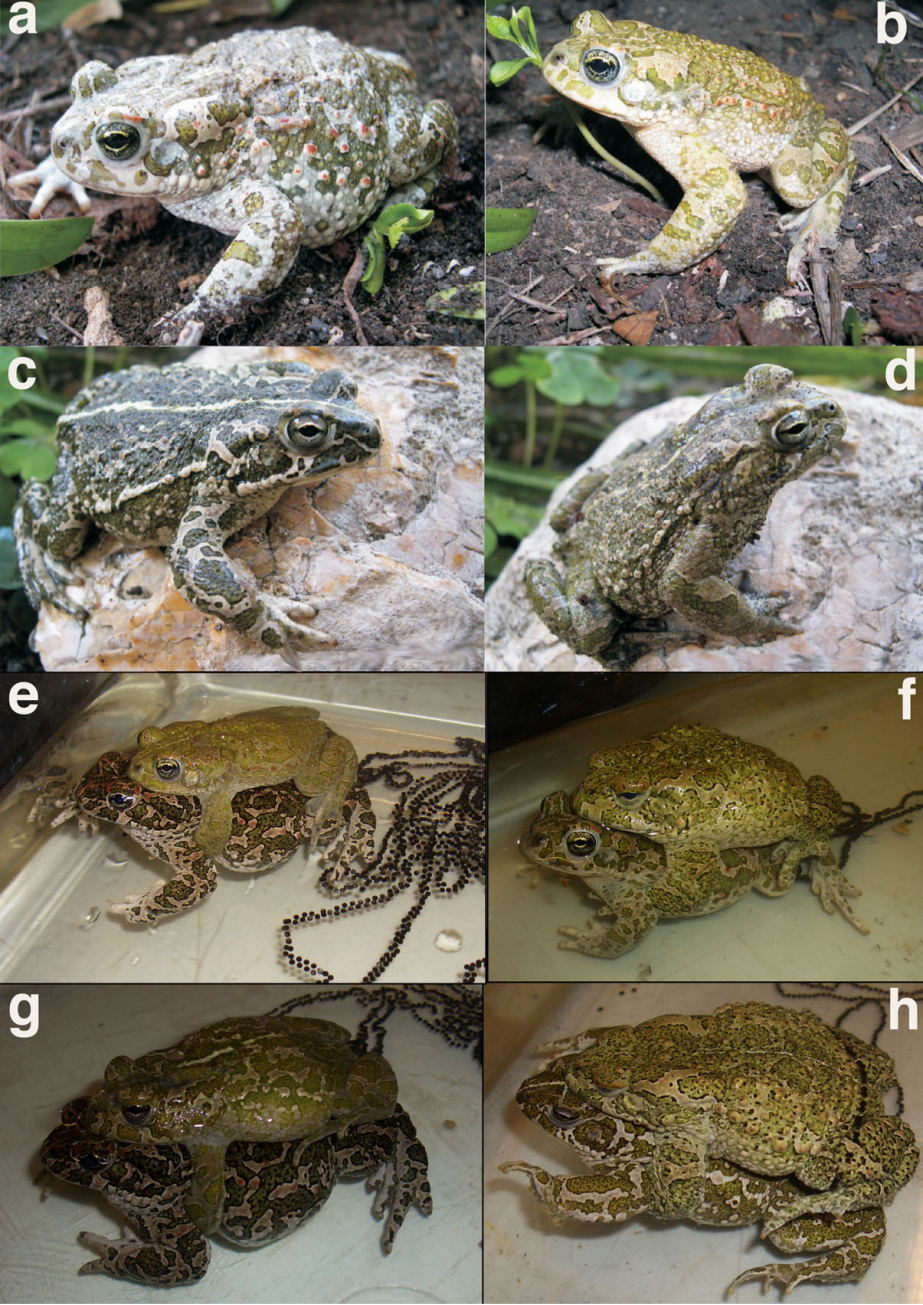
**Backcrosses of F_1 _(*B. balearicus × B. siculus*, Fig. 6a) to parental species of Sicilian green toads**. a-d: Wild-caught animals involved in backcrosses. a: *B. balearicus *female; **b**: *B. balearicus *male; **c**: *B. siculus *female; **d**: *B. siculus *male. **e-h: Backcrosses**. **e**: female F_1 _(*B. balearicus *× *B. siculus*) × male *B. balearicus*; **f**: female *B. balearicus *× male F_1 _(*B. balearicus *× *B. siculus*); **g**: female F_1 _(*B. balearicus *× *B. siculus*) × male *B. siculus*; female *B. siculus *× male F_1 _(*B. balearicus *× *B. siculus*). Photographs: **a-d**: G.F. Turrisi; **e-h**: M. Stöck.

**Figure 7:**
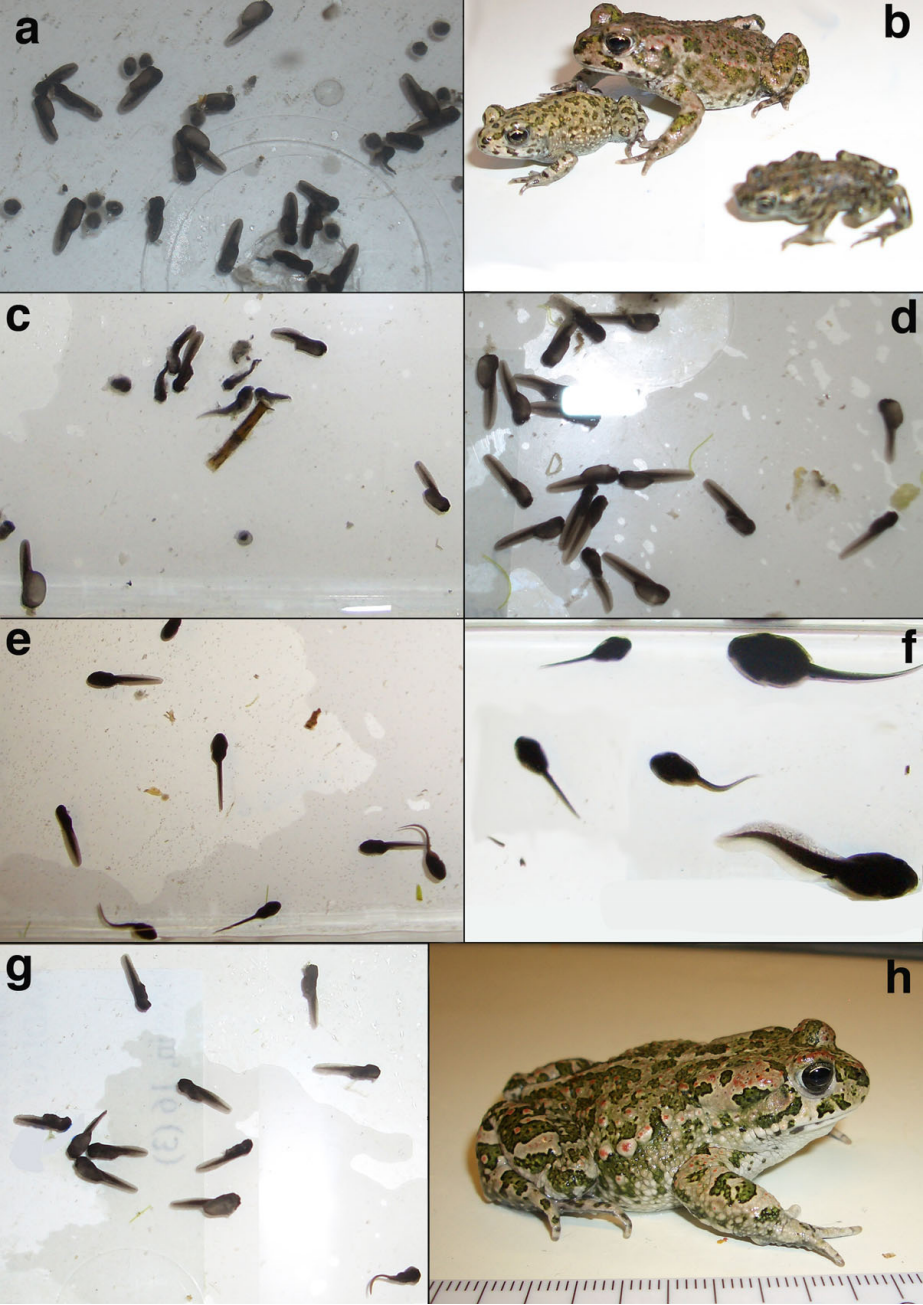
**Survival and development of backcrosses**. a, c to e, g: tadpoles, one week after spawning. a: female *B. siculus *× male F_1 _(*B. balearicus *× *B. siculus*) - note dead embryos and malformations; **b: **postmetamorphic toadlets exhibited size differences among siblings and low survival; **c**: F_2 _from among hybrid crosses, female F_1 _(*B. balearicus *× *B. siculus*) × male F_1 _(*B. balearicus *× *B. siculus*) - all tadpoles malformed; **d: **female F_1 _(*B. balearicus *× *B. siculus*) × male *B. balearicus *- most tadpoles malformed; **e, f: **female *B. balearicus *× male F_1 _(*B. balearicus *× *B. siculus*) - note enormous size differences among siblings in the age of one month after spawning (**f**); **g: **female F_1 _(*B. balearicus *× *B. siculus*) × male *B. siculus - *note most tadpoles show malformations; **h: **F_1_-hybrid from crossing female *B. siculus *× male *B. balearicus *(as shown in Figure 6b) in the age of one year. Photographs: M. Stöck.

We raised about 160 F_1_-metamorphs from the 2007 cross to a snout-vent-length of ca. 2 cm, and kept 50 of them until secondary sexual characters became visible (paler coloration and nuptial pads of males). Though sex ratio was approximately even, we noticed about 30% of dwarfed F_1_-males that reached only about two-thirds the size of normal individuals. Ten males and ten females were further raised until maturity (Figures 5g-h). In spring 2009, we used one F_1 _of each sex to produce F_2_-hybrids (F_1 _× F_1_) and reciprocal backcrosses with either *B. siculus *or *B. balearicus *(one new, wild-caught male and one female each, Figure 6). F_2_-hybrids turned out to be unviable, with all tadpoles dying a few days after hatching. While 200 out of 328 tadpoles from the backcrosses were still alive two months after spawning, they presented, a number of developmental abnormalities, including greenish individuals and a bimodal size distribution within the same cross (Figure 7), and suffered from dramatic mortality at later stages, with two individuals only surviving after metamorphosis.

## Discussion

Our study shows that two distinct lineages of green toads (*Bufo viridis *subgroup) occur parapatrically in Sicily. These lineages have diverged some 2.7 My ago [23], a time frame long enough to allow significant differentiation on both mitochondrial and nuclear DNA sequences (Figure 2). As our study further shows, this divergence was also sufficient to bring the speciation process close to completion.

On the one hand, the endemic *B. siculus*, of North-African origin [home of its sister clade *B. boulengeri*; [23]], occupies the western and southern parts of Sicily, plus two small islands off the north-western coasts (Ustica and Favignana) [23]. On the other hand, *B. balearicus *occupies the north-eastern part of Sicily. Based on their geographical localization and patterns of genetic similarity with mainland Italy, we infer that these Sicilian *B. balearicus *populations recently originated from close-by Calabrian populations. Faunal exchange across the Strait of Messina [including amphibians [29]] are well documented for the Upper Pleistocene [30]. From our genetic analyses, these two species nowadays meet at the eastern coast of Sicily, between the Mount Etna and the Ionian Sea. We cannot exclude that another contact exists along the North coast (north-west of Mount Etna), but could not find any currently occupied site in this area despite thorough examination.

Though very restricted, the documented contact zone shows signs of past hybridization, with differential introgression patterns depending on markers. Mitochondrial alleles show a clear North-South cline, where the frequency of *balearicus *haplotypes progressively decreases from 94% (pop. 13, Calatabiano) to 0% (pop. 16, Misterbianco) over a distance of ca 40 km. Cytonuclear disequilibrium occurred in individuals from both species, pointing to a two-way introgression. This presumably involved symmetric events of hybridization, followed by backcrossing of fertile F_1_-females with their paternal species. Though no F_1_-hybrids were detected in the field, the occurrence of rare and symmetric events of hybridization was confirmed by a few backcrosses (F_2 _or more) identified via STRUCTURE and NEWHYBRIDS in both *B. siculus *and *B. balearicus *populations, as well as a two-way leak of diagnostic nuclear alleles.

However, nuclear introgression was surprisingly low overall. The transition between tropomyosine alleles from the two clades was abrupt, occurring at some point between populations 14 (Giarre) and 15 (Gravina), separated by just 16 km. The same holds for autosomal microsatellites in general, since STRUCTURE assigned (with 100% probability) all individuals from pop. 14 to *balearicus*, and all individuals from pop. 15 to *siculus *(Figure 3). The sharpness of this transition is underlined by the patterns of genetic differentiation: The two populations each side of the contact zone (pop. 14 and 15), though harboring a mix of mitochondrial haplotypes from both lineages, display the highest differentiation value observed in the study area (pairwise F_ST _= 0.52). Populations at the contact zone (clusters b3 and s3) are actually the ones most differentiated on the first PCAGEN factors (Figure 4).

Genetic drift certainly plays a role in this strong local differentiation. The *B. balearicus *populations at the contact zone represent the front wave of a recent expansion, as evidenced by the drastic decrease in genetic diversity from mainland Italy (Hs = 0.74) to the southernmost populations (Hs = 0.38 in population 14). Drift is certainly further amplified by the geographic localization of the contact zone: The geographical bottleneck between Mount Etna and the Ionian Sea creates a peninsular situation, largely isolating populations at the contact zone from conspecifics. This induces a strong differentiation over a small geographic scale, which somewhat blurs the overall clear pattern of isolation by distance observed in both species.

Drift might also partly account for the marked contrast between mitochondrial and nuclear introgression. Mitochondrial markers have low effective sizes (about one quarter of nuclear markers), and are therefore more prone to introgression. In small hybridizing populations, mtDNA might sometimes get fixed into foreign taxa via drift or founder effects, even with little or no gene flow at nuclear loci [31]. This contrast should be further amplified if females display lower effective size than males, which occurs when dispersal is male biased [as expected in polygynous mating system such as found in bufonids; [32-36]]. Effective population sizes at the front wave of expansions (or in any poorly connected population) largely depend on gene flow from incoming immigrants, and thus on the mode of inheritance of markers when dispersal is sex biased [37,38].

In addition to drift and dispersal, selection often contributes crucially to the maintenance of narrow hybrid zones [39]. We think that the one under study is no exception, and that selection against nuclear introgression is very likely to also play a role. First, F_1_-hybrids from our experimental crosses showed reduced fitness (Table 3). Outcomes from reciprocal crosses were asymmetric, which often occurs in interspecific crosses (as documented e.g. in hybrids between green toads and *Bufo calamita *[40] or *B. bufo *[41,42]. If the small size and altered coloration observed in males from the *B. balearicus *× *B. siculus *cross translate into lower survival or fertility in the field, then nuclear markers are indeed expected to show less introgression than mtDNA. Second, survival was drastically affected both in backcrosses and in F_2_-hybrids (F_1 _× F_1_), which should strongly reduce introgression. Third, the currently known ranges of both taxa in Sicily roughly coincide with the borders of semiarid (*B. balearicus*) and arid (*B. siculus*) climates [43]. Adaptation of these two genomes to different climates may select against hybrids, and potentially stabilize the contact zone.

The sex-specific phenotypic effects in the F_1 _as well as the dramatic hybrid breakdown observed both in backcrosses and in the F_2 _generation (F_1 _× F1) were expected from the classical Dobzhansky-Muller model of speciation [44], arising from the confrontation of incompatible genes and the disruption of co-adapted sets of genes. Except for sex chromosomes in the heterogametic sex, F_1_-hybrids inherit complete sets of genes from both parental species and should thus suffer little from co-adaptation losses. We do not know, however, whether sex-specific differences in F_1 _phenotypes (dwarfed males) conform to Haldane's rule [45], because sex-determination mechanisms are unknown for Sicilian green toads (as for most amphibians). Results from related species suggest a XX/XY-system for *B. variabilis *(as "*B. viridis*") from Asia Minor [46,47], but a ZZ/ZW-system for taxonomically undefined green toads from Moldavia [*B. viridis*/*B. variabilis *contact zone?, [48]]. Contrasting with F_1_, backcrosses and F_2 _(F_1 _× F_1_) inherit imbalanced numbers of genes from both parental species due to recombination in F_1_, and may thus lack crucial alleles at complementary loci. Inbreeding presumably also played a role in our F_2 _crosses, which may partially explain the additional mortality relative to the breakdown observed in backcrosses (Table 3). However, inbreeding is also expected to affect F_1 _× F_1 _crosses in the field, given the scarcity of hybridization events. It is also worth noting that a few backcrossed tadpoles survived metamorphosis. Among such rare individuals, females are most likely responsible for the mitochondrial introgression and signs of nuclear allele leakage observed in the field.

Important caveats obviously apply to our crossing experiments, mainly due to the practical difficulties in obtaining reproducing individuals from the field. Absence of replicates limits the power of our inferences, and we lack the exact controls for intraspecific matings (although the latter is compensated by our long experience of breeding green toads in the lab, which allowed us to provide intraspecific controls from other Western Palearctic lineages; Table 3). Results from these preliminary crosses must be clearly considered as provisional, but are worth reporting here as a support for our main conclusions gathered from field population-genetics data.

Though many hybrid zones have been documented in amphibians (see Background), few provide the data required to calibrate the speciation process, in terms of reliable time of divergence and patterns of introgression in the field. A notable exception is provided by fire- and yellow-bellied toads, which constitute one of the best-studied anuran hybrid zones. *Bombina bombina *and *B. variegata*, thought to have diverged during Upper Miocene or Lower Pliocene [3.5 Mya; [49-51]] hybridize in narrow, stable zones maintained by selection and dispersal [51]. Strong selection against hybrids is generated both by hybrid breakdown [21,52], and by environment-dependent selection against toads in mismatched habitats [53].

However, mitochondrial introgression in *Bombina *seems more limited than in Sicilian green toads, with mtDNA clines similar to or even steeper than those of nuclear loci [allozymes; [54]]. The divergence between *B. siculus *and *B. balearicus *is slightly more recent (Plio-Pleistocene, ca 2.7 Mya), but, despite higher mitochondrial DNA introgression, we found almost no admixture at supposedly neutrally evolving nuclear microsatellite loci, suggesting stronger selection to keep gene pools apart.

## Conclusions

While anuran species that diverged > 8 Mya may exhibit partial or complete hybrid inviability in the laboratory, as recently shown for instance by a combination of experimental crosses and molecular divergence time estimates for the *Fejevarya limnocharis *group [55], there is accumulating evidence that anurans with Plio-(Pleisto)cene divergence tend to be reproductively isolated under natural conditions. Secondary contacts between Australian hylids of Plio-Pleistocene divergence have provided evidence for allopatric speciation driven by reinforcement mechanisms, characterized by highly asymmetric F_1_-viability in experimental crosses [8]. Neither F_1 _and F_2_-hybrids nor backcrosses could be identified at a contact zone between two *Hyla *species with an estimated Pliocene divergence (3.6 Mya) [20], indicating a lack of current gene exchange. By contrast, phylogeographic studies [e.g. [16,17]] have shown that clades of more recent divergence [1.33 Mya; [17]] form "wide hybrid zone(s) with a considerable genetic exchange between the two gene pools" [16], suggesting that reproductive barriers are still low or inexistent.

The observed post-mating barriers (hybrid breakdown) in Sicilian green toads certainly induce selection to act against hybridization. Therefore, we expect some pre-mating mechanisms to have evolved since the first contact of these allopatrically evolved lineages, which might also explain the absence of F_1_-hybrids. These two species have already been shown to differ in breeding phenology [23]. Pre-mating isolation between closely related species (through mating calls and female choice) is widespread in anurans, sometimes even in absence of post-mating isolation [56]. The nature of barriers might also affect the structure of hybrid zones, since, due to female choice, mtDNA is expected to introgress more readily than nuclear DNA under pre-zygotic isolation mechanisms (and even more so when the invading species is relatively rare compared to residents) [57]. Further insights on the evolutionary interactions between *B. siculus *and *B. balearicus *populations might be gained by collecting bio-acoustic and mate-choice data, together with additional crossing experiments.

Whatever the exact causes, our data clearly show a virtual absence of gene flow at the present contact zone (corroborated with a hybrid breakdown in backcrosses and F_2_), meaning that the speciation process can be considered as close to have reached complete reproductive isolation after some 2.7 My of divergence. These field data contrast sharply with the results from experimental hybridization in anurans, which show that some lineages may still produce viable F_1 _offspring after ca. 20 My of divergence (see Background). Information on F_1_-hybridizability or viability gained under laboratory conditions may thus grossly overestimate the time required for genetic isolation and speciation to occur in anurans.

## Methods

### Sampling

Samples from 323 specimens of green toads were collected during intense fieldwork between 2004 and 2007 at 24 localities across the Italian Peninsula and Sicily (Figure 1 and Table 1). Italian Peninsula was added to the sampling to allow us to understand the context of the *B. balearicus *invasion in Sicily and to compare Sicilian *B. balearicus *genotypes with those of mainland origin. A few samples came from scientific collections (MVZ, NME, ZFMK) or were collected throughout the years (e.g. road-kills). Tissue samples consisted of finger tips and muscles (road kill) from sub adult and adult toads, and tail tips from tadpoles. Most adults were released at the sampling sites, some vouchers were deposited in institutional collections [MVZ, NME, ZFMK, details Appendix 1 in 23], and tissues were stored in 98% ethanol and/or at -20°C.

### Crossing experiments

Toad crosses were performed with mature adult males and females in a naturally reproductive state during the breeding period in spring. Females were stimulated to spawn by injection of 0.1 ml of a 0.9% NaCl-solution containing 500-1000 IU of human choriogonadotropin (Sigma).

A first crossing experiment was made in the laboratory in 2007 between a female *B. balearicus *(Si 41, pop. 11) and a male *B. siculus *(Si 11, pop. 22), from which 200 F_1_-hybrid tadpoles were raised. Ten randomly chosen offspring were genotyped and added to our sampling ("population 25"). Seven others crossing experiments were made in the laboratory in 2009 using two *B. balearicus *individuals (male: Si 336; female: Si337; Sicily, Marina S. Biagio, pop. 9), two *B. siculus *individuals (male: Si 334; female: Si 335; Sicily, Pergusa Lake, province of Enna, 37°31'00.29" N, 14°18'04.34"E, W of pop. 18 and 19) and two F_1_-hybrid individuals coming from the first crossing experiment (male: Cross 13; female: Cross 11). As no proper control for intraspecific crosses could be performed (due to lack of available females), we provide results from intraspecific matings of other green toad lineages (Table 3: Control 1: *B. turanensis*, Control2: *B. pewzowi*), which were raised under identical conditions.

Crossing pairs from 2009 were observed during amplexus every hour. When 10-15 cm of clutch strings had been deposited, couples were removed from the tank and separated, animals were rinsed to avoid sperm contamination and arranged in new cross combinations in another tank (see Table 3 for cross combinations).

Clutch was left untouched until hatching or until visible signs of dying eggs/embryos were found that had to be removed to avoid a chain-reaction of embryo suffocation. After one month, 100 tadpoles were randomly chosen among the surviving (if so) offspring, raised in shallow oxygenated aquaria and fed with *Elodea *plants and fish food (Tetramin) under identical conditions. After the second month of development, percentage of surviving tadpoles was determined by individual counts. All of these tadpoles were further raised until metamorphosis or death. Toadlets were fed with *Drosophila *and juvenile crickets, and mealworms with weekly additions of calcium and vitamins. Illumination of terrariums included sun-light spectrum fluorescent lamps including a natural-like UV fraction. Clutch, embryonic, tadpole and juvenile development were documented photographically (Figures 5 to 7).

### DNA extraction and microsatellite data generation

DNA was extracted using the Qiagen DNeasy™kit. Microsatellite primer (Bcal μ10) described by Rowe *et al. *[58] and six pairs of microsatellites (BaturaC203, C205, C218, C223, D105, D5) developed by Colliard *et al. *[24] were selected for analysis, due to their level of polymorphism and applicability in both species. PCR-amplification, electrophoresis and allele scoring were performed as described in [24]. Multiplexing of PCR products was performed. For D105 and D5 we used a mixture in a 1:3 ratio; Bcal μ10 was 5% diluted and mixed with C223 and C203 products in a 1:2:2 ratio. The third multiplexing involved products of C218 and C205 in a ratio of 1:2.

### Mitotyping, sequencing and analyses of mitochondrial DNA and nuclear DNA

We used a mitotyping approach described in Colliard *et al. *[24] to detect the mtDNA haplotype group for all individuals. We also sequenced 577 bp of the mitochondrial control region (D-loop) [as described in [18]] in 162 individuals from across the study area (Figure 2).

An intron of *alpha*-Tropomyosine, situated between exons 5 and 6 was amplified, cloned and sequenced as described by Stöck *et al. *[23]. We used homologous sequences from 21 individuals from throughout the sampling area plus one individual from a museum collection coming from a locality near pop. 12. Four of these 22 green toads came from the contact zone with assignment values varying from 68% to 92% to either parental species.

All mitochondrial and nuclear sequences were submitted to GenBank (Acc.-Numbers HM852594-HM852744, in part from [23]). Maximum likelihood (ML) phylogenies of mitochondrial and nuclear sequence alignment were generated using PhyML version 2.4.5 [59] and using HKY models according to MrModeltest [60]. In each case, we choose a BioNJ as a starting tree, and optimized topology, branch length and rate parameters. Other parameters were used as defaults of the program. We generated bootstrap values based on 1000 resampled data sets.

### Genotype data analyses

To exclude genotyping errors due to null alleles, stuttering and allelic drop-out, Micro-Checker v.2.2.3 [61] was used. Genotypic linkage disequilibrium between each pair of loci per population was tested using ARLEQUIN v. 3.0 [62]. Significance was adjusted for multiple tests using Bonferroni corrections. Estimation of heterozygote deficits and its significance was assessed using FSTAT v. 2.9.4 [63]. For each microsatellite marker and species, we estimated genetic diversity by calculating number of alleles (Na), and within-sample expected heterozygosity (Hs) (Additional file 1).

Isolation by distance was investigated with Mantel tests (FSTAT) by regressing pairwise F_ST_/(1-F_ST_) against the natural logarithm-transformed Euclidean geographic distances [64], for *B. balearicus *and *B. siculus *independently, selecting localities with ten or more samples. Two sets of analyses were performed, including or not the three populations at the contact zone which showed mitochondrial introgression. A principal component analysis was conducted with PCAGEN [28] to visualize pairwise differentiation among populations (F_ST_), with 1000 randomizations of genotypes to test for significance of axes.

In order to better determine potential signs of introgression between the two species, we used the Bayesian clustering algorithm of STRUCTURE v. 2.2 [25]. We used the admixture model and allowed for correlated allele frequencies between populations, as recommended by the authors for cases of subtle population structure. We tested a range of cluster numbers (K) from 1 to the number of localities per analysis, plus an additional three to enable us to potentially infer subtle structure. Each run, replicated 10 times, consisted of 10^5 ^iterations, after a "burn-in" of 10^4^. To infer which K best fits our data, we applied the ad hoc ΔK statistic developed by Evanno *et al. *[26]. We performed three analyses: (1) all Sicilian localities plus the lab-cross individuals; (2) all *B. siculus *localities, and (3) all *B. balearicus *localities.

### Identification of hybrids

Four alternative approaches were used to identify hybrids. First, we used the cluster assignment value (STRUCTURE, parameters as above, K = 2), considering as hybrids all individuals with assignment values below 90%. Second, we performed two analyses using NEWHYBRIDS v.1.1 Beta3 [27] to assign Sicilian toads to genotypic classes (parental, F_1_, F_2_, backcrosses). The method computes Bayesian posterior probability that an individual belongs to each of these different hybrid classes while simultaneously estimating allelic frequencies for parental species. Runs were repeated several times with varying lengths of the "burn-in" and number of sweeps, as recommended in the program manual. The first analysis was based on all Sicilian individuals. The second focused on populations where hybrids were shown to occur or were likely to do so (pop. 12 to 16 and 18), with addition of few pre-assigned non-hybrid individuals from "pure" populations (pop. 9 to 11 and 17), using the z option as recommended by the authors [27]. Third, we identified individuals as hybrids if they were assigned by STRUCTURE to one clade but contained mtDNA from the other (= cyto-nuclear disequilibrium). Finally, we compared the microsatellite allele composition of Sicilian *B. siculus *and *B. balearicus *populations far apart from the contact zone (i.e., presumably pure) with those closer to the contact zone (potentially admixed). Alleles found in presumably pure populations that were exclusively present in one species were considered as "diagnostic". Finding diagnostic alleles of one clade in individuals assigned to the other clade by STRUCTURE was considered a sign of past hybridization (Additional file 3).

Research has been carried out according to approved guidelines under the following permits: Regione Siciliana, Assessorato Agricoltura e Foreste Prot. No 89884, Palermo, Italy; Bundesamt für Veterinärwesen BVET Nr. 791/09, Bern, Switzerland; and Authorisation No. 1798, Service de la consommation et des affaires vétérinaires, Canton de Vaud, Epalinges, Switzerland.

## List of abbreviations

MtDNA: mitochondrial DNA; My: million years: Mya: million years ago; nuDNA: nuclear DNA; pop.: population; MVZ: Museum of Vertebrate Zoology, University of California, Berkeley, USA; NME: Naturkundemuseum Erfurt, Germany; ZFMK: Zoologisches Forschungsinstitut und Museum Alexander Koenig, Bonn, Germany.

## Authors' contributions

The fieldwork was conducted by AS, CC, GFT and MSt, and the molecular work (genotyping, mitotyping and sequencing) by AS, CC and MSt. The crossing experiments were performed by CC and MSt, and the statistical analyses and data interpretation by CC, MSt, MA and NP. CC drafted the paper, which was edited by MSt and NP, then completed by AS, GFT and MA. All authors read and approved the final manuscript.

## Supplementary Material

Additional file 1 **Table with allele size ranges, number of alleles, within-species expected heterozygosity (H_S_), Weir and Cockerham (1984) estimator of inbreeding coefficient for each locus and each species**. Click here for file

Additional file 2 **Table with posterior probabilities for different genotypic classes (parental, F_1_, F_2 _or backcrosses) for the four wild-caught individuals identified as possible F_2_-hybrids, using the program NEWHYBRIDS**. Click here for file

Additional file 3 **Table with distribution of alleles from all seven microsatellite loci in the twenty-six potential hybrids, and summary of STRUCTURE assignment and mtDNA haplotype groups**. Click here for file

Additional file 4 **Table with pairwise F_ST _per pair of populations and their respective significance**. Click here for file
