# Supplementary material for: Strong reproductive barriers in a narrow hybrid zone of West-Mediterranean green toads (Bufo viridis subgroup) with Plio-Pleistocene divergence
Source: BMC Evol Biol. 2010 Jul 29;10:232. doi: 10.1186/1471-2148-10-232 (PMC2923517; doi:10.1186/1471-2148-10-232)
Supplement: Additional file 1 — Table with allele size ranges, number of alleles, within-species expected heterozygosity (HS), Weir and Cockerham (1984) estimator of inbreeding coefficient for each locus and each species. [file 1471-2148-10-232-S1.PDF]

Additional file 1 – Allele size ranges (in base pairs), number of alleles (Na), within-species expected heterozygosity (H_S), Weir and Cockerham (1984) estimator of inbreeding coefficient (F_{IS}) for each locus and each species. Significance levels are indicated as follows: * < 0.05, ** < 0.01, and *** < 0.001.

**(A)** ***B. balearicus***				
**Locus**	**Size range (bp)**	**Na**	H_S	F_{IS}
**Bcalμ10**	149-161	5	0.35	0.013
**BaturaC203**	195-215	6	0.45	0.055
**BaturaC205**	158-186	8	0.63	0.033
**BaturaC218**	155-187	9	0.65	-0.029
**BaturaC223**	163-195	9	0.53	-0.075
**BaturaD105**	144-208	11	0.70	0.049
**BaturaD5**	122-152	8	0.73	0.008
**Overall**		8.43	0.58	0.002
**(B)** ***B. siculus***				
**Bcalμ10**	159-185	11	0.60	0.106*
**BaturaC203**	199-227	5	0.33	0.015
**BaturaC205**	160-268	19	0.77	0.058
**BaturaC218**	139-179	10	0.75	0.088
**BaturaC223**	167-199	6	0.62	-0.017
**BaturaD105**	145-196	7	0.67	0.064
**BaturaD5**	114-215	18	0.64	0.205***
**Overall**		10.86	0.63	0.079***
