# Supplementary material for: Strong reproductive barriers in a narrow hybrid zone of West-Mediterranean green toads (Bufo viridis subgroup) with Plio-Pleistocene divergence
Source: BMC Evol Biol. 2010 Jul 29;10:232. doi: 10.1186/1471-2148-10-232 (PMC2923517; doi:10.1186/1471-2148-10-232)
Supplement: Additional file 2 — Table with posterior probabilities for different genotypic classes (parental, F1, F2 or backcrosses) for the four wild-caught individuals identified as possible F2-hybrids, using the program NEWHYBRIDS. [file 1471-2148-10-232-S2.PDF]

Additional file 2 – Posterior probability of assignment to different genotypic classes (parental, F_1, F_2 or backcrosses) for the four wild-caught individuals identified as possible F_2-hybrids, using the program NEWHYBRIDS.

**Individual**	**Posterior probability for each genotypic class (%)**					
	B. balearicus	*B. siculus*	F_1-hybrid	F_2-hybrid	Backcross with *B. balearicus*	Backcross with *B. siculus*
**Si140** (pop. 13)	27.3	0	0.004	66.3	6.4	0.003
**Si185** (pop. 13)	22.2	0	0	74.6	3.2	0
**Si187** (pop. 18)	0	42.2	0	55.8	0	2
**Si196** (pop.18)	0	39.5	0	59.7	0	0.8
