# Supplementary material for: Strong reproductive barriers in a narrow hybrid zone of West-Mediterranean green toads (Bufo viridis subgroup) with Plio-Pleistocene divergence
Source: BMC Evol Biol. 2010 Jul 29;10:232. doi: 10.1186/1471-2148-10-232 (PMC2923517; doi:10.1186/1471-2148-10-232)
Supplement: Additional file 3 — Table with distribution of alleles from all seven microsatellite loci in the twenty-six potential hybrids, and summary of STRUCTURE assignment and mtDNA haplotype groups. [file 1471-2148-10-232-S3.PDF]

Individual	Population	STRUCTURE assignment	mtDNA haplotype	D5 Allele 1	D5 Allele 2	D105 Allele 1	D105 Allele 2	C223 Allele 1	C223 Allele 2	Bcalµ10 Allele 1	Bcalµ10 Allele 2	C203 Allele 1	C203 Allele 2	C205 Allele 1	C205 Allele 2	C218 Allele 1	C218 Allele 2
**Si185**	13	*B. balearicus*	*B. balearicus*	122	122	184	192	163	167	153	153	199	199	170	170	163	167
**Si143**	13	*B. balearicus*	*B. siculus*	148	152	196	196	163	175	153	153	199	199	170	174	163	163
**Si140**	13	*B. balearicus*	*B. balearicus*	135	144	188	192	167	175	149	149	199	215	158	170	167	171
**Si167**	14	*B. balearicus*	*B. siculus*	148	148	196	196	163	167	153	156	199	199	170	174	163	167
**Si165**	14	*B. balearicus*	*B. siculus*	148	148	196	196	163	167	153	153	199	199	170	170	163	163
**Si163**	14	*B. balearicus*	*B. siculus*	148	148	176	196	167	167	156	156	199	199	170	170	167	175
**Si151**	14	*B. balearicus*	*B. siculus*	148	148	192	196	163	167	153	156	199	199	174	174	163	175
**Si150**	14	*B. balearicus*	*B. siculus*	148	148	196	196	163	167	153	153	199	199	174	174	163	163
**Si149**	14	*B. balearicus*	*B. siculus*	148	148	196	196	163	167	153	153	199	199	170	174	163	167
**Si152**	14	*B. balearicus*	*B. siculus*	148	148	196	196	163	167	153	153	199	199	174	174	163	163
**Si145**	14	*B. balearicus*	*B. balearicus*	148	148	176	176	167	167	153	156	199	199	170	170	167	175
**Si24**	15	*B. siculus*	*B. balearicus*	199	199	188	188	183	199	165	165	199	223	160	160	159	171
**Si23**	15	*B. siculus*	*B. balearicus*	144	199	180	188	183	183	163	165	219	223	220	248	171	171
**Si135**	15	*B. siculus*	*B. balearicus*	144	199	180	188	183	187	163	165	223	231	160	220	171	171
**Si133**	15	*B. siculus*	*B. balearicus*	199	199	180	192	183	199	165	171	219	223	168	220	155	171
**Si132**	15	*B. siculus*	*B. balearicus*	199	199	188	192	183	199	163	171	219	223	160	168	139	171
**Si131**	15	*B. siculus*	*B. balearicus*	144	199	188	188	183	183	165	171	199	223	168	220	139	155
**Si126**	15	*B. siculus*	*B. balearicus*	199	207	180	180	183	183	165	167	219	223	160	168	155	159
**Si125**	15	*B. siculus*	*B. balearicus*	199	199	180	188	183	183	163	167	219	223	160	248	155	155
**Si122**	15	*B. siculus*	*B. balearicus*	199	199	188	188	183	183	165	171	199	223	160	168	NA	NA
**Si121**	15	*B. siculus*	*B. balearicus*	199	199	188	192	183	183	163	165	219	223	160	248	171	171
**Si120**	15	*B. siculus*	*B. balearicus*	199	199	188	188	183	187	163	165	199	223	160	248	171	171
**Si119**	15	*B. siculus*	*B. balearicus*	144	199	188	188	183	183	165	171	199	223	168	248	155	155
**Si115**	15	*B. siculus*	*B. balearicus*	199	203	180	192	183	183	169	171	199	223	160	160	155	171
**Si187**	18	*B. siculus*	*B. siculus*	144	148	184	192	183	183	159	159	199	199	160	168	163	163
**Si196**	18	*B. siculus*	*B. siculus*	148	152	184	188	183	183	159	167	199	219	172	216	139	171

Additional file 3 – Distribution of alleles from all seven microsatellite loci in the twenty-six suspected hybrids, and summary of STRUCTURE assignment and mtDNA haplotype groups. Green alleles are *B. balearicus* diagnostic alleles and orange alleles are *B. siculus* diagnostic alleles. White alleles are common to both *B. balearicus* and *B. siculus*. Individuals highlighted in yellow correspond to individuals showing at least one allele from the other species than assigned by STRUCTURE.
