# Supplementary material for: Strong reproductive barriers in a narrow hybrid zone of West-Mediterranean green toads (Bufo viridis subgroup) with Plio-Pleistocene divergence
Source: BMC Evol Biol. 2010 Jul 29;10:232. doi: 10.1186/1471-2148-10-232 (PMC2923517; doi:10.1186/1471-2148-10-232)
Supplement: Additional file 4 — Table with pairwise FST per pair of populations and their respective significance. [file 1471-2148-10-232-S4.PDF]

Additional file 4 – Pairwise F_{ST} between populations (below diagonal) and their respective significance (above diagonal). Significance levels are indicated as follows: * < 0.05, ** < 0.01, and *** < 0.001.

Population	5	6	8	9	10	13	14	15	16	17	18	21	22	23
**5**		*	***	***	***	**	***	***	***	***	***	***	***	***
**6**	0.05		***	***	***	***	***	***	***	***	***	***	***	***
**8**	0.2	0.16		***	***	***	***	***	***	***	***	***	***	***
**9**	0.25	0.23	0.12		***	***	***	***	***	***	***	***	***	***
**10**	0.18	0.13	0.11	0.07		***	***	***	***	***	***	***	***	***
**13**	0.21	0.17	0.14	0.18	0.11		***	***	***	***	***	***	***	***
**14**	0.39	0.28	0.24	0.3	0.26	0.12		***	***	***	***	***	***	***
**15**	0.3	0.31	0.39	0.39	0.35	0.4	0.52		***	***	***	***	***	***
**16**	0.26	0.28	0.31	0.31	0.26	0.32	0.46	0.3		***	***	***	***	***
**17**	0.18	0.2	0.27	0.27	0.23	0.27	0.41	0.16	0.1		***	***	***	***
**18**	0.18	0.2	0.26	0.26	0.21	0.25	0.41	0.17	0.09	0.02		**	***	***
**21**	0.2	0.23	0.26	0.26	0.21	0.27	0.4	0.23	0.12	0.09	0.07		***	***
**22**	0.33	0.34	0.36	0.38	0.31	0.38	0.52	0.39	0.24	0.21	0.16	0.11		***
**23**	0.31	0.32	0.35	0.36	0.32	0.36	0.51	0.28	0.19	0.18	0.16	0.16	0.32	
